# G. T. Myles

**Published:** 1939

**Authors:** 


### Obituary

### GEORGE THOMAS MYLES, M.R.C.S., L.R.C.P.

George Thomas Myles, who died on 17th November, 1939, was intimately associated with Bristol, being a son of the Rev. James Perceval Myles, who was Vicar of St. Matthias, Bristol, before becoming Rector of West Kington.

Myles entered the Bristol Medical School when its premises were in Park Row. He was contemporary with Paul Bush, who later became his brother-in-law. He was a keen sportsman, playing Rugby regularly for the Medical School and being well-known in first-class Rugby circles.

He was a student at the Bristol Royal Infirmary and a strong supporter and admirer of this institution. He qualified in 1884, and was appointed House Physician to the Bristol Royal Hospital for Sick Children and Women.

Starting professional work in the centre of the city, he speedily built up a large general practice, and in 1899 moved into Clifton, where he continued at work until his retirement in 1938.

He was a man of great personal charm, and one who never spared himself in the service of his patients. He was a fine clinician and one of the best of family doctors. He was a strong opponent of the National Health Insurance Act when it was first brought forward, and he played a prominent part in the opposition to the Panel system.

His chief hobby was gardening, and his flowers, notably his dahlias and chrysanthemums, bore evidence of his characteristic energy and success.

Dr. Myles leaves a widow, a daughter and a son who has succeeded him in his practice. We offer to them our deep sympathy.

